# HPV Vaccines: Separating Myths from Reality

**DOI:** 10.1055/s-0039-1693691

**Published:** 2019-07

**Authors:** Julio César Teixeira, Cecília Maria Roteli-Martins

**Affiliations:** 1 Universidade Estadual de Campinas, Campinas, SP, Brazil; 2 Center of Clinical Research, Faculdade de Medicina do ABC, Santo André, SP, Brazil

It is widely known that human papillomavirus (HPV) infection is sexually transmitted and can range from benign lesions such as genital warts to anogenital and oropharyngeal cancer.

In the United States, among men and women, 33,700 individuals are diagnosed with HPV-associated cancer each year. Vaccination could prevent ∼ 90% of these cases, which means that 31,200 cases could be avoided each year.^1^

In Brazil, numbers from the Cancer Registries and of the Mortality Information System (SIM/MS, in the Portuguese acronym) for the year 2018, estimated the incidence of 16,370 new cases of cervical cancer, with 5,000 deaths. This rate has remained stable in recent decades, despite screening and prevention efforts.^2^

Among HPV-associated cancers, such as in the penis, the vagina, the vulva, the anus and in the oropharynx, the only type with screening recommendations is cervical cancer, which is performed by gynecologists. Thus, except for the cervix, cancers in other sites are usually detected after the development of symptoms.^3^

The association of periodic screening and HPV vaccination is the primary strategy for eliminating cervical cancer. The HPV vaccine has been recommended for all preadolescents and adolescents since 2011 by the World Health Organization (WHO). In Brazil, since 2014, this vaccine is offered by the Brazilian Unified Health System (SUS, in the Portuguese acronym) for girls between 9 and 14 years old, and, as of 2017, also for boys. From 2020, the ages for vaccination of girls and boys will be the same, starting at 9 years old.

Two doses of the vaccine are required to ensure vaccine protection, and they should be applied at a 6- to 12-month interval. Individuals between 9 and 26 years old living with HIV or with some immunodepressive conditions also receive the vaccine by the SUS. In this specific case, in 3 doses applied at 0, 2, and 6 months.^4^

Since 2014, the 1^st^ dose of HPV vaccine was administered in between 50 and 61% of the Brazilian population of girls aged 9 and 10 years old, and the second dose in only between 22 and 38% of this population, which is below the target of 80% coverage established by the National Program of Immunizations (PNI, in the Portuguese acronym).^5^

The HPV vaccine coverage of up to 80% can also prevent precursor lesions, and consequently reduce the need for screening tests and invasive treatments, exhausting follow-up and future obstetric harm.^6^ ^7^

If HPV vaccines are available from the SUS, why has optimal vaccine coverage not been achieved?

Since 2007, when vaccination began in Australia, and until now, several events or situations that temporarily hamper the expansion of the use of these vaccines have been repeated around the world. Among these situations, are some unfounded concepts that vaccination would stimulate early sexual activity or reduce the use of other protective measures for safe sex. Although the hepatitis B vaccine also has the sexual transmission factor, it does not present rejection or problems and is universally accepted.^8^

Another situation that interferes with vaccination coverage rates in Brazil is related to the concern with the safety of the vaccines available. The most common adverse events of HPV vaccines are transient and similar to symptoms related to other vaccines, such as pain at the site of injection, headache, dizziness, and syncope. Syncope is a characteristic situation of vaccination in preadolescents and in adolescents, who are targets of these vaccines.^9^ ^10^

The Brazilian Ministry of Health (MS, in the Portuguese acronym) clarifies the safety and effectiveness of the HPV vaccine. Like all vaccines offered by the PNI, there is a strict process of validation and registration with the National Agency of Sanitary Surveillance (ANVISA, in the Portuguese acronym). However, like any medicine, it can cause postvaccine adverse events, although they are rare.

The HPV vaccine is being used in > 80 countries around the world, so far without any reported evidence linking its use to severe or neurological conditions. In 2017, the WHO Vaccine Safety Commission released a document reaffirming the safety of these vaccines after the administration of 270 million doses.^5^ ^11^

Although the safety of these vaccines is confirmed by scientific evidence and by epidemiological surveillance, unexpected situations causing some level of anxiety in the population have occurred all over the world. In Brazil, during a school-based vaccination campaign in the city of Bertioga, state of São Paulo, in 2016, there were reports of “paralysis” in 11 girls after the 2^nd^ dose of the vaccine. The fact was widely reported in the press and generated a negative highlight regarding vaccination. However, the immediate actions of the surveillance and vaccination authorities in the state of São Paulo and the subsequent follow-up of the cases confirmed that this was a transient reaction, possibly related to the anxiety of the adolescents. There were no sequelae, and the causal relationship with the HPV vaccine used was ruled out. Although these are enlightening facts, they were not properly released in the media.

This imbalanced priority of bad and negative news, coupled with the reverberation of past messages through social media as if they were ‘current’, has helped to maintain a continuous negative effect on the population, interest of anti-vaccine groups, which affects vaccine coverage.

More recently, in 2018, the press reported events possibly related to vaccination in a group of girls in the city of Rio Branco, state of Acre, Brazil. The PNI sent a team to Acre and, after evaluating each case together with a local team, most of them had no causal or temporal nexus, such as menstrual irregularity, ovarian cyst, appendicitis, leptospirosis, or cytomegalovirus, and were initially discarded. Among the remaining cases, conversion disorders stood out, some with diagnoses prior to vaccination. Two cases with seizures continued to be monitored by the local team and by neuropediatricians, one with known cerebral vascular malformation and the other under investigation.

Due to the news spread from local media, ‘new’ cases started to emerge in Acre and, apparently, there were difficulties and lack of objectivity in local actions for the clarification of the diagnoses. Thus, part of the population remains unsure about vaccination, while other people involved appear to have secondary gains with the persistence of this situation. The local media foments the situation with some sensationalism. The result is a worrying and substantial drop in vaccination rates in Acre, which even reached the inadmissible point of state doctors not indicating vaccination.

In conclusion, with the information collected so far in Acre, there is no confirmed association between the cause and the HPV vaccination performed; hence, vaccination should continue.

## Procedure in Case of Refusal to Vaccinate

Many parents and guardians of adolescents have refused vaccination against HPV. This refusal has been a phenomenon observed with other vaccines as well. For physicians facing this situation, it is advisable to provide information on the safety of vaccines and reasons not to delay vaccination. If parents or guardians decide not to apply the vaccine, it should be registered in the medical record that the vaccine was prescribed and refused. Physicians should continue to discuss the benefits of immunization in subsequent visits, because some parents may reconsider their decision not to vaccinate or simply postpone vaccination. Reasons for refusal to vaccinate should be addressed by all professionals.

## Importance to Vaccinate Now

The choice of some parents or guardians to not vaccinate increases the risk for everyone. Only one adolescent that initiates his sexual activity without vaccination protection and acquires the infection can transmit it to all partners, even for those vaccinated, but who did not achieve sufficient levels of antibodies for protection. Vaccination with close to 100% coverage increases safety for everyone and the hope that we can eradicate cases of HPV-related cancer in a few decades.

Finally, all tools for controlling HPV-related cancers are known and available, and there is no safety problem with HPV vaccines so far.

In Brazil, the use of these tools must evolve, because they are already available and require no additional investments, only organization and clear direction. A simple and logical suggestion is to start with cervical cancer through routine HPV vaccination by using the winning strategy of school-based vaccination and achieve a population registration system that allows advances toward the organization of the national screening program.
